# Editorial: Commending 20 years since the formal discovery of immune priming: the innate immune memory

**DOI:** 10.3389/fimmu.2025.1652666

**Published:** 2025-08-27

**Authors:** Jorge Contreras-Garduño, Joachim Kurtz

**Affiliations:** ^1^ Escuela Nacional de Estudios Superiores, unidad Morelia, National Autonomous University of Mexico, Morelia, Mexico; ^2^ Institute for Evolution and Biodiversity, University of Münste, Münster, Germany

**Keywords:** ecology, ecological immunology, adaptive immunity, trained immunity, immune priming, evolution, specific immune memory, parasites

For many decades, the prevailing paradigm has been that only vertebrates, but not invertebrates, possess immune memory. However, already since the 1970s, studies in insects and crustaceans suggested that organisms previously exposed to a sublethal pathogenic challenge could better survive a following lethal challenge. Exposed individuals were also shown to have enhanced immune responses, and to more effectively eliminate pathogens, compared to those not pre-exposed (1–6). Nevertheless, these responses often lacked specificity, which is considered a hallmark of immune memory. Thus, the belief in the absence of immune memory in invertebrates largely prevailed until it was reported that immune memory in invertebrates could show specificity to a degree that it could differentiate between siblings and unrelated parasite lines (7). This sparked considerable debate among the scientific community regarding the capacity for immune memory in invertebrates (8, 9), and over time, a growing number of studies have revealed innate immune memory in various invertebrate groups (10). Moreover, an increasing focus on immune memory provided by cells of the innate immune system of vertebrates led to the discovery of “trained immunity” (11). Today, “trained immunity” usually refers to innate immune memory in vertebrates, while the term “immune priming” is normally restricted to invertebrates. While highlighting potential mechanistic differences between these forms of innate immune memory (12, 13), this distinction may also limit the exchange of ideas. Interactions between researchers in the fields of trained immunity and immune priming thus bear large potential. Therefore, 20 years after the inaugural studies of “immune priming”, we initiated this Research Topic to gather articles that provide further evidence and review the status of immune priming and trained immunity, aiming to assess future directions.

Several articles in this Research Topic provide further evidence for immune priming and analyze conditions determining its occurrence. Immune priming has previously been suggested to be contingent on parasite virulence, the evolutionary costs of immune memory and/or the host immune response performance (14). Goerlinger et al. now propose that the route of infection is also an important determinant, at least, in insects (*Tenebrio molitor*) against bacteria, and they also reveal another interesting result: the host behavior seems to be very important during immune priming. These are promising research avenues for future investigations.

Recent studies highlight that immune priming carries physiological costs. Cortacans et al. demonstrate that *Drosophila melanogaster* primed and challenged with *Candida albicans* strain 4372 exhibited a strong antimicrobial peptide (AMP) response, with sex-specific immune pathway activation (Toll in males, Imd in females). However, this enhanced response did not improve survival, suggesting potential costs of immune overactivation. Similarly, Cime-Castillo et al. show that in mosquitoes, heterologous priming with different Dengue virus serotypes (e.g., DENV-4 then DENV-2) reduced pupation rates but, in some cases, increased adult emergence. These results suggest a trade-off between development and immune activation, with implications for vector control and transgenerational immunity, though further generational data are needed.

Sułek et al. contribute to important evidence regarding mechanistic underpinnings of immune priming, which can vary strongly among different systems. They found that immune-primed *Galleria mellonella* larvae upregulated a peptide (Pr13a) that reduced bacterial load and altered pathogen surface properties, pointing to potential effector molecules involved in immune memory. Cho and Cho report that *Gryllus bimaculatus* primed with heat-killed *Bacillus thuringiensis* exhibit increased survival and extracellular trap formation, with evidence that immune protection becomes more specific over time. As far as we know, this is the first study reporting that extracellular traps are involved in immune priming. Collectively, these findings underscore that immune priming may not be universally beneficial and is shaped by host-pathogen interactions, immune effectors, and timing. Long-term studies are essential to fully understand the dynamics and costs of invertebrate immune memory.

While the precise mechanisms by which organisms recognize specific immune challenges, establish immune memory, and recall it across successive encounters remain incompletely understood (15), the studies featured in this Research Topic make a valuable contribution by proposing a novel interplay between metabolism, epigenetics, and the endocycle in shaping immune memory (Méndez-López et al.; Mukherjee and Dobrindt). This integrative perspective advances the field by highlighting potential molecular and cellular frameworks underlying immune priming and its long-term maintenance. Accordingly, Ng et al. in this Research Topic made use of the same copepod-tapeworm system where specificity was first demonstrated in invertebrate immune memory to identify differential molecular mechanisms of specific versus non-specific immune priming. A transcriptomic approach pointed to epigenetics and metabolism associated with both forms of priming, while splicing-associated processes were characteristic of specific priming and oxidative phosphorylation and carbon metabolism of unspecific priming. Importantly, epigenetics and metabolism are also involved in trained immunity (16), so, these might be general mechanisms underlying innate immune memory in animals. Accordingly, Boraschi et al. provide an important conceptual and comparative approach to both trained immunity and immune priming. They argue that for both types of immunity it depends on the challenges and conditions whether innate memory is non-specific or specific, and whether it is long- or short-lived. Vertebrate innate immune memory can also show some degree of specificity, likely mediated by receptors and pathway involved in the initial recognition process.

Within the scope of trained immunity, the authors contributing to this Research Topic propose several potential applications. Sui and Berzofsky suggest leveraging trained immunity to enhance cancer immunotherapies, while Samuel et al. propose the use of BCG vaccination to stimulate trained immune responses in bovines. However, Bhargavi and Subbian caution the scientific community about the potential drawbacks associated with the development of non-specific vaccines. Specifically, they highlight that inflammation induced by trained immunity may contribute to the emergence of autoimmune pathologies. In light of these concerns, the authors emphasize the importance of carefully designing novel therapeutic strategies for both infectious and non-infectious diseases. Their review focuses on the immunologic, metabolic, and epigenetic mechanisms underlying trained immunity, particularly within myeloid cells. Furthermore, evidence from invertebrate models suggests that immune priming may incur evolutionary costs, particularly in terms of reproductive fitness (17). Given these considerations, the potential costs and trade-offs of immune priming and trained immunity warrant further investigation, especially in contexts where their application has been proposed.

The articles in the Research Topic highlight important features of innate immune memory that may direct future research. The field of immune priming–initially driven by input from evolutionary ecology–has now identified a multitude of mechanistic underpinnings in the diverse organisms studied. Acknowledging this diversity allows for comparisons with vertebrate trained immunity, where knowledge of mechanisms is already very rich, while avoiding oversimplistic generalizations. Future research may include evolutionary aspects to evaluate consequences of applications of trained immunity and immune priming in human and animal health and beyond.
